# Treatment of Aggressive Pituitary Adenomas: A Case-Based Narrative Review

**DOI:** 10.3389/fendo.2021.725014

**Published:** 2021-11-15

**Authors:** Odelia Cooper, Vivien Bonert, Ning-Ai Liu, Adam N. Mamelak

**Affiliations:** ^1^ Pituitary Center, Department of Medicine, Cedars-Sinai Medical Center, Los Angeles, CA, United States; ^2^ Department of Neurosurgery, Cedars-Sinai Medical Center, Los Angeles, CA, United States

**Keywords:** pituitary adenomas, surgery, radiation therapy, temozolomide, targeted therapy, aggressive adenomas

## Abstract

Management of aggressive pituitary adenomas is challenging due to a paucity of rigorous evidence supporting available treatment approaches. Recent guidelines emphasize the need to maximize standard therapies as well as the use of temozolomide and radiation therapy to treat disease recurrence. However, often these adenomas continue to progress over time, necessitating the use of additional targeted therapies which also impact quality of life and long-term outcomes. In this review, we present 9 cases of aggressive pituitary adenomas to illustrate the importance of a multidisciplinary, individualized approach. The timing and rationale for surgery, radiation therapy, temozolomide, somatostatin receptor ligands, and EGFR, VEGF, and mTOR inhibitors in each case are discussed within the context of evidence-based guidelines and clarify strategies for implementing an individualized approach in the management of these difficult-to-treat-adenomas.

## Introduction

Pituitary adenomas are benign, slow-growing tumors that typically respond to standard surgical and medical therapies, and only ~0.2% become malignant (1, 2). However, there is an intermediate stage, in which benign pituitary adenomas follow a more aggressive clinical course. Although they do not metastasize, these adenomas may be large and may be invasive, showing Knosp scores of 3-4 and invasion of the sphenoid sinus and other vital structures. They also may recur at early timepoints and do not respond to multiple therapies, with >20% exhibiting persistent growth despite optimal medical, surgical, and radiotherapy (3). In these patients, escalated treatment strategies beyond standard therapies are required to control continued tumor growth and prevent tumor-associated local and/or systemic morbidities.

Overall, patients harboring aggressive adenomas require multidisciplinary focused expert care by a team dedicated to pituitary disorders (4). In this review, we present 9 cases of aggressive pituitary adenomas treated at the Cedars-Sinai Pituitary Center, a tertiary referral center with a multidisciplinary care team in line with the recommended structure for a Pituitary Tumor Center of Excellence (4). We defined aggressive adenomas as radiologically invasive adenomas with unusually rapid growth rate or clinically relevant growth despite optimal standard therapies (3). We aim to illustrate the importance of a multidisciplinary, individualized treatment approach. We discuss these cases within the context of current evidence-based guidelines, highlighting critical issues in determining the appropriate time to intervene and in weighing patient- and disease-specific factors that influence treatment selection.

## Summary of Cases 

The 9 patients with aggressive adenomas included 7 males and 2 females, ranging in age from 20 to 56 years, diagnosed with 3 silent corticotroph adenomas (SCAs) that later converted to Cushing disease (CD); with one becoming a carcinoma, 1 CD; 1 null cell carcinoma; and 4 prolactinomas, of which one progressed to a pituitary carcinoma. There were no cases of aggressive growth hormone (GH)-secreting adenomas;. Initial adenoma size ranged from 19 to 90 mm (**Table 1**). Pathologic features are described in **Table 2**. Of note, SF-1 immunostaining was not available (**Table 1**).

**Table 1:** Summary of cases.

Case	Year of Dx	Age at Dx	Sex	Type	Features*	Recurrences	Initial therapy	Surgery	RT	Medical therapy	Follow-up	Disease duration
1	1997	45	M	SCA→CD	25 mm, right cavernous sinus invasion	6	TSS	1997 TSS	1998 GK SRS	2013: TMZ x 7 cycles: PR	2017: Transformed to carcinoma with cerebellar and cervical spine metastases	21 yrs
								2012 TSS	2010 CK SRS	2013: Pasireotide x 1yr: SD		
								2015 TSS	2015 FRT	2017: Bevacizumab x 3 mo: PD		
									2016 Proton SRS		2018: Deceased	
2	2010	45	F	Null cell	19 mm, left cavernous sinus invasion	2	TSS	2010 TSS	2011 IMRT	2011: TMZ x 9 cycles: PD	2012: Developed *de novo* orbital metastasis, consistent with transformation to carcinoma	10 yrs
								2011 TSS	2012 IMRT	2012: Lapatinib x 2 yrs: no recurrence		
								2012 TSS				
											2020: No tumor	
3	2012	29	M	PRLoma	90 mm; left cavernous invasion, mass effect on brainstem and 4th ventricle.	5	Cab with maximum dose of 10.5 mg/week	2014 CRX	2019 FRT	2019: TMZ x 6 cycles: SD	2020: Stable residual tumor with no evidence of metastases	8 yrs
								2016 TSS				
								2017 TSS				
								2019 TSS				
4	2001	24	F	SCA→CD	59 mm; invasion of right cavernous sinus, Meckel’s cave and prepontine cistern	6	CRX	2001 CRX	2002 GK SRS	2014: TMZ x 4 cycles: SD	2020: Decreased tumor size, ACTH 134 with no evidence of metastases	19 yrs
								2002 TSS	2015 FRT/TMZ	2014: Pasireotide x 2 mo: PD		
								2007 CRX		Ketoconazole: PD		
								2008 CRX		Metyrapone: PD		
								2014 BLA		Mitotane: PD		
										2016: Cabergoline: SD		
										Pasireotide: SD		
5	2004	40	M	SCA→CD	24 mm; right cavernous sinus invasion, encasing carotid	2	TSS	2004 TSS	2013 RT	2015: TMZ x 6 cycles: PR	2019: No visible tumor and no evidence of metastases	15 yrs
								2013 TSS	2015 RT/TMZ	2016: Bevacizumab x 6 mo		
								2015 CRX				
6	1996	56	M	CD	28 mm; clival invasion	3	TSS	1997 TSS	2000 RT		2015: No sellar tumor	18 yrs
								1998 TSS				
								2000 TSS			New dural masses consistent with meningiomas	
								2013 TSS				
7	1994	54	M	PRLoma	40 mm; in anterior foramen, right infratemporal fossa, occipital condyle	3	Cab with maximum dose of 14 mg/week	1996 TSS	1996 GK SRS	2006: TMZ x 2 cycles: PD	2006: Developed metastatic disease to the skull base, neck, and lymph node, consistent with carcinoma	12 yrs
								2005 TSS				
											Deceased	
8	2009	20	M	PRLoma	40 mm; invasion of bilateral cavernous sinuses, Meckel’s cave	1	Cab with maximum dose of 10.5 mg/week	2021 TSS		2017: TMZ x 6 cycles: PR	2021: Tumor growth (pre-TSS) with no sign of metastatic disease	12 yrs
9	2008	36	M	PRLoma	40 mm; bilateral cavernous sinuses	3	Cab with maximum dose of 12 mg/week	2013 TSS	2015 RT	2020: Octreotide	2021: Decreased tumor size with no sign of metastatic disease	13 yrs
								2014 TSS		2020: TMZ x 7 cycles: PR		
								2019 CRX				

*Features of recurrent adenomas are listed if data were not available from initial presentation.

ACTH, adrenocorticotropin; BLA, bilateral adrenalectomy; Cab, cabergoline; CD, Cushing disease; CK, CyberKnife; CRX, craniotomy; Dx, diagnosis; FRT, fractionated radiation therapy; GK, GammaKnife; IMRT, intensity-modulated radiation therapy; PD, progressive disease; PRL, prolactin; PRLoma, prolactinoma; PR, partial response; RT, radiation therapy; SCA, silent corticotroph adenoma; SD, stable disease; SRS, stereotactic radiosurgery; TMZ, temozolomide; TSS, transsphenoidal surgery; Yrs, years.

**Table 2:** Pathologic data.

Case	Pathologic adenoma type	Ki-67	Mitotic count	p53
1	Corticotroph	5%	Not increased	Weakly positive
2	Null cell	10%	Many mitotic figures	positive
3	Densely granulated lactotroph	0.5%	Not increased	60% positive (1-2+)
4	Null cell (initially)	Moderately high	Not increased	N/A
5	Densely granulated corticotroph	20%	rare	80% positive
6	Densely granulated corticotroph	1%	Not increased	< 0.5%
7	Lactotroph	N/A	N/A	N/A
8*	N/A	N/A	N/A	N/A
9	Densely granulated lactotroph	9%	Not increased	< 0.1% (1+)

*Patient underwent TSS for drainage of cyst. No viable tumor was visualized or resected; pathologic analysis therefore is not available (N/A).

The 4 patients with prolactinomas were treated initially with the dopamine agonist (DA) cabergoline, and the remaining adenomas were treated initially with surgery. Recurrences were defined as growth of the residual adenoma after subtotal resection or *de novo* recurrence after gross total resection. One patient developed a *de novo* recurrence while the remainder experienced residual adenoma growth. Eight of the 9 patients had more than one recurrence: 2 had 2 recurrences, 3 had 3 recurrences, 1 had 5 recurrences, and 2 had 6 recurrences. Time to first recurrence ranged from 1 to 9 years, with 4 patients exhibiting recurrence at 1 year, 2 patients at 2 years, and 1 patient each recurring at 5 years and 9 years. Duration of disease ranged from 8 to 21 years; 2 patients are deceased.

## Maximizing Standard Medical Therapy for Functioning Adenomas

For patients with apparently aggressive functioning adenomas, standard medical therapies should be optimized to ensure that continued adenoma growth is not due to under-dosing (3). For prolactinomas, cabergoline should be administered at a dose of least 3.5 mg a week, and PRL normalization has been reported with doses as high as 11 mg a week (5, 6). For CD, pituitary-directed therapy with the SRL pasireotide can be attempted to decrease ACTH and control adenoma growth (7). When maximal doses have been unsuccessful in controlling disease, further treatment options should be considered.

In the cases presented here, the 4 patients with prolactinomas received maximally tolerated doses of cabergoline ranging from 10.5 to 14 mg a week, although the adenomas continued to progress and PRL levels remained elevated, necessitating additional therapeutic interventions. In one case of an SCA that converted to florid CD, the patient received several medications, including ketoconazole, metyrapone, and mitotane, but hypercortisolism remained uncontrolled and she ultimately underwent bilateral adrenalectomy. The other CD patients underwent surgical resection of the recurrent adenoma without receiving prior medical therapy.

## Surgical Resection

MRI evidence of total adenoma removal after initial surgery correlates with long-term disease-free survival in more than 90% of patients among patients with non-functioning adenomas (NFAs) (8–10). Even when residual tumor tissue is visible on postoperative MRI, approximately 60% to 70% of adenomas will show little evidence of regrowth over a period of approximately 10 years (11, 12); in these cases, regrowth occurs at a rate of approximately 1 mm per year. For functioning adenomas, recurrence of hormonal hypersecretion can occur in up to 25% of patients with CD and up to 40% of patients with prolactinomas at 5 years (13–15), for which re-operation or medical therapy is often effective.

By contrast, for patients with refractory adenomas, surgery plays a lesser role (16–19). These adenomas may invade the cavernous sinus, skull base, or intracranial structures beyond the sella and parasellar region, reducing the opportunity for gross total resection, and there is high likelihood of further growth even with extensive resection. On initial presentation, adenomas are more likely to have a soft texture and a pseudo-capsule, which is a thin, non-fibrotic band of tissue that surrounds and encapsulates the tissue, separating the tumor from the normal gland and other structures, allowing for “extracapsular” removal of the entire mass. However, recurrent adenomas are far more fibrotic and poorly encapsulated (20, 21), which, when combined with previous surgical scarring, makes surgical debulking far more challenging. Nonetheless, particularly for rapidly growing adenomas, reoperation can be considered to reduce mass effect, i.e., to decompress critical structures such as the pituitary gland or optic apparatus, in anticipation of subsequent radiation therapy (RT) or chemotherapy (22).

In general, there is no absolute limit to the number of times transsphenoidal surgery or craniotomy can be attempted, although potential benefits are typically diminished after 3-4 surgeries due to progression of tumor into areas in which surgical resection is difficult or tumor texture renders meaningful debulking impossible.

Among the 9 cases of aggressive adenoma, 4 patients underwent 3 surgeries, 3 had 4 surgeries, 1 patient had 2 surgeries, and 1 underwent surgery after progression on temozolomide (TMZ). While surgery was successful in debulking the adenoma in all cases, gross total resection was not achieved, and the residual adenoma continued to grow. This pattern of subtotal resection and growth of postoperative residual adenoma is typical of aggressive adenomas.

## Radiation Therapy

RT has proven reliable when surgical resection is deemed not feasible or when medical therapy is no longer efficacious or tolerated (23–26). Treatment doses in the range of 12-15 Gy delivered in a single fraction or in multiple fractions can prevent further adenoma growth in more than 95% of adenomas. However, rates of radiation-induced hypopituitarism are quite significant, with at least 25% and up to 90% of patients developing a degree of anterior pituitary hormone deficiency within 10 years (25, 27, 28). In addition, other factors such as patient age, symptoms, adenoma aggressivity, and adenoma type must be taken into consideration when weighing treatment benefit and timing. RT also can be used to reduce hormone secretion in nonresectable functioning adenomas that have failed to respond to medical therapy or that demonstrate feedback-induced adenoma growth (e.g., Nelson’s syndrome in patients with progressive CD) (29–31).

A role for repeat irradiation of adenomas that progress or recur despite previous RT is far more limited due to substantial risk of radiation necrosis to adjacent brain structures or breakdown of the carotid artery wall leading to catastrophic bleeding or death (32, 33). The overlap between new and old radiation treatment fields as well as the time between radiation treatments should be considered when determining the potential risk-benefit ratio of additional RT. Improved planning software, more accurate treatment devices, and better understanding of adenoma physiology has allowed for lower radiation dose, which, in turn has resulted in reduced long-term secondary risk such as cognitive deterioration or carotid injury. Nonetheless, the absolute dose tolerance of brain and surrounding structures typically limits use to one or two sessions.

Eight of our 9 patients had RT immediately after initial surgery or after multiple surgeries: 4 patients had a single course, 3 had 2 courses, and 1 had 4 courses. Five patients initially responded to RT, demonstrating either stable disease or some decrease in size, while the remaining 3 patients had continued progressive disease despite RT. All patients showed disease recurrence/progression over time.

## Temozolomide

TMZ demonstrates clinical efficacy in up to one-third of aggressive adenomas and its use in these cases is widely reported (34). TMZ is recommended for aggressive pituitary adenomas that grow despite surgery, RT, and optimized medical therapy (3). TMZ may also be considered in frail patients who may not tolerate repeat surgery or have relative contraindications to RT (35). However, the timing of treatment initiation, the duration of therapy, and the potential additive benefits of combining TMZ with RT or another agent all remain unknown.

The standard treatment schedule for TMZ monotherapy is 150-200 mg/m^2^/day for 5 days every 4 weeks. Regimens that administer lower TMZ doses for longer periods in a cycle, such as 50 mg/m^2^ daily for 21 days (36), have been associated with increased myelotoxicity (37). In patients where the combination of TMZ with RT is considered, such as in those with rapidly growing adenomas with elevated Ki-67 >10%, extensive p53 expression, and/or high mitotic count (3), TMZ can be initiated at lower doses during and then escalated after RT. For example, treatment may be administered as 6 weeks of continuous TMZ therapy at 75 mg/m^2^ daily with RT, followed by 6-12 months of standard TMZ monotherapy at 150 mg/m^2^ daily for 5 days every 28 days and increasing as tolerated to 200 mg/m^2^ (38). Among 166 patients with aggressive pituitary adenomas treated by 67 surveyed members of the European Endocrine Society (ESE), 14 patients received concomitant TMZ and RT with complete or partial response observed in 71% compared to 34% in those on TMZ monotherapy (39), suggesting the efficacy of combination therapy.

Treatment efficacy with TMZ is typically evident by 3 months (40), although many patients may not see maximal adenoma response until 6 months (39). Of note, biochemical response often parallels response on MRI, and may even improve further once adenoma growth stabilizes (39, 41). In the ESE survey, complete radiologic response, i.e., no visible adenoma, was observed in 6% and complete biochemical response in 19% of cases, while partial radiologic response with >30% reduction in size was seen in 31% and partial biochemical response with >20% hormone reduction in 24% of cases (39). A systematic review of TMZ in aggressive pituitary adenomas and carcinomas showed similar response rates, with 6.5% of 31 aggressive adenomas achieving complete response, 45% partial response, 29% stable disease, and 19% progressive disease, while 8.7% of 23 pituitary carcinomas showed complete response, 61% partial response, 13% stable disease, and 17% progressive disease (42).

TMZ therapy is usually administered for 6 months (3), although case reports describe up to 12 months of treatment. Median treatment duration of 9 months in the ESE survey was associated with treatment response (39). A study of 30 adenomas treated with at least 6 cycles of TMZ showed that 27% had adenoma regression, 47% stable disease, and 27% adenoma progression. After a medial follow-up of 34 months, 40% maintained stable disease and 60% had progression (41).

Longer duration of TMZ treatment has been shown to be beneficial in maintaining disease-free progression up to 120 months (42). However, it can be difficult to quantify because reports describe widely varying treatment durations. In a multicenter study, patients treated for more than 12 months with TMZ had a median relapse-free survival of 57 months versus 18 months for those treated for 12 months or less (43), suggesting the benefit with “longer-term” therapy is seen after 12 months. By contrast, the systematic review of 31 aggressive adenomas and 23 carcinomas defined “short-term” TMZ treatment as a median of 8 months (range, 1-12 cycles) and “long-term” as a median of 26 months (range, 14-45) cycles. Among patients who responded, 36% were receiving long term TMZ; 5-year progression-free survival rate was numerically but not significantly different at 61.3% for long-term TMZ compared to 16.3% for short-term TMZ, but overall survival rate was significantly improved with long-term therapy (91.7% vs 54.1%; p=0.08). Of note, 95% of patients had received prior RT and 75% were previously treated with other standard therapies (42). In a report of 8 patients with aggressive adenomas treated with TMZ for more than 12 months, 4 patients had partial response and the other 4 had stable disease; response was maintained while on TMZ. Of the 3 patients with ACTH-secreting adenomas, one showed complete remission of hypercortisolism, one with Nelson’s syndrome showed >90% reduction in ACTH level, and the third showed ~40% reduction in ACTH and urinary free cortisol (44).

Based on available reports, it is reasonable to consider continued TMZ therapy beyond 6 months in patients who are responding to treatment.

The combination of TMZ with the oral chemotherapy capecitabine in the CAPTEM protocol has shown some efficacy in aggressive CD. In a report of 4 cases, 2 had complete adenoma resolution, 1 stable disease, and 1 a 75% decrease in adenoma size concomitant with ACTH reduction (45); another reported case of CAPTEM in an aggressive CD patient showed radiologic stable disease and biochemical response (39). However, use of CAPTEM in 2 corticotroph carcinomas and one lactotroph carcinoma were not successful (39, 40).

MGMT status has been proposed as a predictor of response to TMZ therapy. MGMT (O6-methyl guanine DNA methyl transferase) is a DNA repair enzyme that negates the effect of TMZ (34) and thus tumoral expression of MGMT is inversely related to TMZ efficacy (40, 46–48). However, patients with high MGMT expression may still respond to TMZ (36, 43, 49); in the ESE survey, 76% of those with low MGMT expression responded, while 46% with high MGMT expression did not (39). Therefore, TMZ should still be considered, regardless of MGMT status (3). Functioning aggressive adenomas have been shown to respond better to TMZ compared to NFAs (45% vs 17%), although the rationale is unknown (39).

A second course of TMZ is usually less effective than the initial course and response has not been consistently demonstrated (39, 43, 50). However, it may be reasonable to attempt a 3-month rechallenge in patients with late relapse and low MGMT, as a report of 9 adenomas treated with a second course of TMZ showed that late relapse after the first course was associated with improved response (34).

Eight of the 9 cases presented here were treated with TMZ. Three patients with SCA that progressed to CD were treated with 4-6 cycles of TMZ, but response varied widely, likely due to both MGMT status and prior/concurrent treatments. One patient with 95% MGMT expression showed radiologic progression after 2 cycles of TMZ. However, the addition of pasireotide enabled biochemical and radiologic response, and he completed 7 cycles of combination treatment before showing signs of relapse. The second patient underwent RT followed by 6 cycles of TMZ and experienced both biochemical and radiologic improvement. MGMT in this patient was negative. The third patient, with an unknown MGMT status, showed both biochemical and radiologic response after 2 cycles but progressed again after 4 cycles. Of 4 patients with prolactinoma, one with an unknown MGMT status showed disease progression after concurrent administration of TMZ and RT followed by TMZ monotherapy for 1 cycle. However, the other 3 patients showed sustained biochemical and radiologic response to 6 cycles of TMZ; MGMT was 1% positive in 2 cases and unknown in the other. Finally, 1 patient with a null cell adenoma underwent RT and 9 cycles of TMZ before the disease progressed. MGMT at the initial surgery and upon removal of an orbital metastasis was 98% positive.

Thus, of 8 patients treated, 2 had initial response but progressed after 4-6 cycles of TMZ, 1 did not respond at all, and 4 had partial response to TMZ.

## SRL Therapy

Clinical response of aggressive pituitary adenomas to SRLs is variable. Somatostatin receptor subtype (SST) expression and differences in SRL affinity for each SST may provide some insight into the likelihood of response. Corticotroph adenomas primarily express SST5, and octreotide and lanreotide, which have a strong affinity for SST2, are largely ineffective (51). Pasireotide, which has a stronger affinity for SST5, has been shown to normalize cortisol levels in 20-40% of CD patients (51). However, reports of aggressive CD treated with pasireotide show minimal effect (39). Of the reported 22 aggressive adenomas treated with pasireotide, only 4 (18%) adenomas showed adenoma response and 5 (23%) showed biochemical response (39, 50, 52). In a report of 3 patients with aggressive ACTH-secreting macroadenoma or carcinoma, 2 showed paradoxical increase in ACTH and urinary free cortisol with pasireotide treatment (53), while another report of 3 patients with recurrent CD after cessation of TMZ showed no effect of pasireotide (50). However, one case of an SCA showed a 6% adenoma volume decrease with TMZ and a 21% decrease after adding pasireotide, and a CD patient showed biochemical response to pasireotide 12 months after partial response to TMZ (52). Some reports show pasireotide reduced ACTH secretion and adenoma size in some patients with Nelson’s syndrome (54), but others show no response (55) or mixed response, with 5 patients in one series showing minimal impact on adenoma volume with pasireotide even though ACTH levels decreased (56).

Pasireotide was administered in 2 SCA cases that progressed to CD. In 1 patient it was added to TMZ and enabled adenoma stabilization for a year. In the second patient, it was added after bilateral adrenalectomy and subsequent RT, and has maintained stabilization of both adenoma volume and ACTH levels for 4 years.

NFAs primarily express SST3 (57). Although octreotide and lanreotide may inhibit cell proliferation in cultured NFAs (58), clinical efficacy is limited. In 39 patients with residual adenoma tissue, 81% of those with positive octreotide uptake on scintigraphy demonstrated stable adenoma volumes with octreotide LAR treatment, but none had adenoma reduction (59). In a review of 100 NFAs in 11 published studies treated with octreotide for an average of 6 months, 12% showed adenoma reduction, 83% stable residual, and 5% increased volume (60). Although pasireotide has a higher affinity for SST3 and has been shown to inhibit NFA cell viability *in vitro* (61), no clinical studies have confirmed control of adenoma growth. Of note, there was no clear association between SST expression and clinical features of aggressiveness in 113 NFAs, of which 46% were invasive (62).

Prolactinomas show poor clinical response to SRLs and treatment of cultured DA-resistant adenomas did not show reduced prolactin (PRL) levels (63). However, clinical improvement has been demonstrated in some patients with confirmed SST5 expression. In 5 patients with DA-resistant prolactinomas, treatment with octreotide LAR and cabergoline reduced PRL levels in 2 patients, with a 93% reduction in adenoma size; SST5 expression was noted in 1 responder (64). Others reported PRL and/or adenoma response in prolactinomas treated with combination cabergoline and pasireotide, including in an aggressive prolactinoma strongly positive for SST5 that demonstrated PRL normalization after 2 months that was maintained for 31 months, with >50% reduction in adenoma size (65), as well as in a cabergoline-resistant giant silent prolactinoma showing high expression of SST5 that demonstrated adenoma shrinkage with pasireotide treatment (66). However, among 47 macroprolactinomas, SST2 and SST5 were expressed in only 3/23 and 3/21 adenomas, respectively, and 1 adenoma with both subtypes (66).

Of the 4 prolactinomas in the set, 1 showed continued adenoma growth and rising PRL levels despite cabergoline dose up to 7 mg/day, multiple surgeries, and RT. Although SST5 expression was negative, he was started on octreotide LAR 30 mg monthly in an attempt to stabilize PRL levels, but PRL remained elevated and adenoma size increased.

Peptide receptor radionuclide therapy (PRRT), an SRL-based therapy in which radiolabeled SST-binding molecules target SST2 and SST5, has been evaluated in aggressive pituitary adenomas, demonstrating octreotide uptake on PET/CT. Although 50% of NFAs, 38% of prolactinomas, and 50% of CD adenomas demonstrated positive uptake with ^111^In-octreotide scintigraphy (67), only 3 of 20 aggressive pituitary adenomas treated with PRRT, showed a partial response and 3 stable disease (reviewed in [68)].

## EGFR-, VEGF-, and mTOR-Targeting Therapy

Another target for aggressive pituitary adenomas is EGFR, which belongs to the ErbB family of membrane receptor kinases (69). To date, 3 EGFR tyrosine kinase inhibitors (TKIs), gefitinib, lapatinib, and canertinib, have been evaluated in the treatment of pituitary adenomas. Gefitinib selectively inhibits EGFR/ErbB1 activity, lapatinib prevents activation of EGFR/ErbB2, and canertinib is an experimental pan-ErbB receptor TKI (70).

EGFR is expressed in 75% of human corticotroph adenomas (69, 71), and gefitinib treatment of human primary pituitary corticotroph tumor cultures reduced POMC mRNA and reversed features of hypercortisolemia (71). Lapatinib treatment of corticotroph tumor cells decreased POMC mRNA and ACTH levels. Canertinib, which targets multiple ErbB receptors, suppressed POMC mRNA and ACTH secretion in cultured human corticotroph adenomas, with a 70% reduction seen in those with higher ErbB expression (72).

Gain-of-function mutations in *USP8* have been implicated in aberrant EGFR signaling in corticotrophs (73–75) and studies show *USP8* mutations in 21-62% of corticotroph adenomas (76). However, it remains unclear whether *USP8* mutations and EGFR overexpression are associated with more aggressive adenomas (73, 75, 77–79). Whole exome sequencing of corticotroph adenomas enriched with aggressive adenomas identified *USP8* mutations in only 5 of 22 adenomas (80), but another study found EGFR expression in the cytoplasm of 29 of 52 CD adenomas, and protein expression associated with recurrence (81). There are as yet no reports of EGFR-targeting therapy in patients with aggressive CD.

The strongest evidence supporting use of these agents is for aggressive prolactinomas. EGFR, ErbB2, and ErbB3 are all endogenously expressed in rat lacto-somatotroph tumor cells, and *in vitro* treatment with EGFR TKIs was shown to prevent PRL secretion (69, 82, 83). GH3 cell lines transfected with a constitutively active form of ErbB2 showed a 250-fold induction of PRL, while treatment of these cells with lapatinib led to 40% suppression of PRL secretion (82). In Wistar-Furth rats inoculated with these same transfectants, treatment with lapatinib led to a 40% suppression of adenoma growth and 50% decrease in PRL levels (82), and Fischer rats implanted with estrogen pellets to recapitulate an endogenous prolactinoma model developed pituitary adenomas with hyperprolactinemia that were suppressed by 35% with lapatinib treatment (82). More than 50% of prolactinomas express EGFR and 25% express ErbB2 protein (reviewed in (69), with 40% of invasive adenomas staining positive for ErbB2, compared to 1.2% of noninvasive adenomas (84). However, rates of ErbB receptor expression were even higher in 28 human prolactinoma specimens analyzed (85), with approximately 80% expressing ErbB receptors, including EGFR (82%), ErbB2 (92%), ErbB3 (25%), and ErbB4 (71%). Higher ErbB3 receptor expression correlated with higher rates of optic chiasm compression, suprasellar extension, and encasement of the carotids, while higher ErbB4 was observed in adenomas with sphenoid sinus invasion (85).

In a 6-month proof-of-concept clinical trial, patients with DA-resistant prolactinomas were treated with lapatinib. One subject demonstrated near normalization of PRL and a 22% reduction in adenoma volume after showing continued adenoma growth and PRL elevation on cabergoline 7 mg/week and surgical adenoma resection; a second subject with persistent hyperprolactinemia and adenoma growth on cabergoline 7 mg/week and surgical resection achieved a 42% reduction in PRL and tumor size stabilization (85).

In a follow-up phase IIa trial, 3 of 4 patients with aggressive prolactinomas treated with 6 months of lapatinib showed stable radiologic disease, with 1 subject showing up to 17% decrease in diameter and a 42% decrease in PRL (86). EGFR expression did not correlate with response to lapatinib therapy.

The 1 patient with a null cell adenoma was treated for 2 years with lapatinib following surgery and RT for an orbital metastasis to help prevent recurrence. After 8 years, she continues to show no signs of recurrence. Her adenoma was positive for EGFR and ErbB2 on immunohistochemistry.

VEGF, expressed in pituitary adenomas, stimulates angiogenesis and regulates the tumor microenvironment to contribute to pituitary tumorigenesis (43, 87, 88). However, treatment of aggressive pituitary adenomas with bevacizumab, a monoclonal antibody binding VEGFR, has yielded mixed results. The ESE survey reported adenoma stabilization in 1 of 3 patients on bevacizumab monotherapy, with another showing partial adenoma regression after combination therapy with TMZ, while a third showed adenoma progression (39). Treatment with bevacizumab after multiple prior therapies including temozolomide in 2 patients with corticotroph adenomas showed decreased ACTH after the first cycle but no longer-term benefit, while 1 patient with NFA showed disease stabilization for 18 months (89).

VEGF-targeting agents in combination with RT and TMZ demonstrated stabilization of a silent corticotroph carcinoma (90), as well as prolonged recurrence-free survival after treatment with RT, TMZ, and bevacizumab (91); progression-free survival for 8 years was observed after RT, TMZ, and bevacizumab in a corticotroph carcinoma with CNS metastases (92). Treatment of 17 pituitary carcinoma patients who recurred after TMZ use also showed some response to bevacizumab monotherapy or combination therapy, including 1 patient with a null cell carcinoma who remains on bevacizumab (93).

Bevacizumab was attempted in 2 patients in our series, both of whom have SCAs that progressed to CD. One had failed multiple courses of RT and showed spinal and dural metastases when he was started on bevacizumab. After 3 months on therapy, he developed cognitive decline, transitioned to hospice, and died. The other patient was started on bevacizumab after undergoing 3 surgeries and 2 courses of RT as well as TMZ. Treatment was given for 6 cycles and he remains recurrence-free 15 years after his initial diagnosis. VEGF expression status was unknown.

The mTOR inhibitor everolimus, approved for the treatment of neuroendocrine tumors, exhibits antiproliferative and proapoptotic activity in pituitary adenomas (94). Clinical response to everolimus is variable, with reports describing partial response in a prolactinoma and stable disease in a corticotroph carcinoma, but progressive disease in 4 other cases of aggressive adenomas (39, 95–97) (**Table 3**).

**Table 3:** Summary of responses to targeted medical therapies.

Therapy	N	Tumor response	Biochemical response
EGFR-targeting gefitinib, lapatinib, canertinib	8	25% (n=2) decrease	38% (n=3)
		50% (n=4) stable	
VEGF-targeting bevacizumab	15	26% (n=4) stable	25% (n =2)
mTOR-targeting everolimus	7	14% (n=1) partial;	
		14% (n=1) stable	

Data from McCormack et al, 2018 (39), Cooper et al, 2021 (86), Osterhage et al, 2021 (89), Ortiz et al, 2012 (90), Touma et al, 2017 (91), Donovan et al, 2016 (96), and Zhang et al, 2019 (97).

## Summary of Treatment Options

Treatment of aggressive pituitary adenomas is challenging. The rarity of the disorder, heterogeneity of aggressive adenomas, the lack of consistent response to treatment options, and limited experience on when and how to initiate each treatment modality all underscore the need to individualize management. The cases presented here illustrate the importance of applying an individualized, multidisciplinary approach within the context of current evidence-based guidelines (**Table 4**).

**Table 4:** Summary of treatment options.

Treatment option	Clinical Considerations
Surgery	Total gross resection and/or adenoma debulking preferred where feasible No upper limit on number of surgeries, although scarring and adenoma location and texture may limit benefits after 3-4
RT	Typically highly effective in controlling nonresectable adenoma growth For repeat irradiation, careful planning is required to avoid injury to surrounding structures
TMZ	Recommended for adenoma that grow despite surgery, RT, and optimized medical therapy Most common regimens: standard dose for 6 cycles if monotherapy, lower dose if given with RT Rechallenge after progression is typically less effective Unclear whether MGMT status is useful in predicting response Longer duration of therapy may sustain response
SRL	Octreotide and lanreotide typically not effective Pasireotide has limited effect in aggressive CD, may be effective in prolactinoma Unclear whether SST5 expression is useful in predicting response
EGFR-targeting	Best evidence for efficacy is with lapatinib in prolactinoma Optimal timing of initiation and treatment duration are unclear
VEGF-targeting	Bevacizumab shows mixed results Combination with RT and/or TMZ may be effective for pituitary carcinomas or some aggressive CD converted from SCA

EGFR, epidermal growth factor receptor; CD, Cushing disease; MGMT, O6-methylguanine-DNA methyltransferase; PRLoma, prolactinoma; RT, radiation therapy; SRL, somatostatin receptor ligand; SST, somatostatin receptor; TMZ, temozolomide; VEGF, vascular endothelial growth factor.

Surgical resection and repeat adenoma debulking are recommended where feasible, especially when mass effects are evident. However, rapid re-growth of residual adenoma tissue requires use of additional therapy. RT, although not curative, may stabilize adenoma growth for months to years, with eventual progression. TMZ is effective in most patients, but responses may be partial and not sustained. Repeat treatment with TMZ is often less effective. Other medical therapies, including SRLs and EGFR-, VEGF-, and mTOR-targeting agents, may stabilize adenoma size and maintain biochemical control in patients with functioning adenomas, but evidence for this is limited. It is unclear whether initiation of these therapies before adenoma growth is evident may help delay progression, allowing TMZ therapy to be ‘reserved’ when deemed clinically necessary. Identification of actionable molecular targets in aggressive adenomas may further help determine whether other kinase inhibitors and checkpoint inhibitors can be of additional benefit (80, 98). Importantly, regardless of whether an adenoma is defined as aggressive, some subtypes may show aggressive behavior and may warrant use of aggressive treatment strategies. These include SCA that evolve to clinically apparent CD, and lactotroph tumors in males that show resistance to dopamine agonist therapy. Continued study of aggressive pituitary adenomas is essential to establishing a treatment paradigm that maintains adenoma control while optimizing patient quality of life.

## Author Contributions

OC and AM conceptualized the project, performed the literature search and data analysis, and drafted the article. OC, VB, and NAL contributed case discussions. All authors contributed to the article and approved the submitted version.

## Conflict of Interest

The authors declare that the research was conducted in the absence of any commercial or financial relationships that could be construed as a potential conflict of interest.

## Publisher’s Note

All claims expressed in this article are solely those of the authors and do not necessarily represent those of their affiliated organizations, or those of the publisher, the editors and the reviewers. Any product that may be evaluated in this article, or claim that may be made by its manufacturer, is not guaranteed or endorsed by the publisher.
